# Comparisons of Effective Fields of Two Ultra-Widefield Ophthalmoscopes, Optos 200Tx and Clarus 500

**DOI:** 10.1155/2019/7436293

**Published:** 2019-12-05

**Authors:** Yoshitsugu Matsui, Atsushi Ichio, Asako Sugawara, Eriko Uchiyama, Hitomi Suimon, Hisashi Matsubara, Masahiko Sugimoto, Kengo Ikesugi, Mineo Kondo

**Affiliations:** Department of Ophthalmology, Mie University Graduate School of Medicine, Tsu, Japan

## Abstract

**Purpose:**

To compare the effective fields of the Optos 200Tx® and Clarus 500™, two ultra-widefield ophthalmoscopes, based on their ability to image branches of retinal vessel in the four retinal quadrants.

**Methods:**

Ninety retinal images from 90 patients with various eye diseases were studied. All patients had undergone 200° retinal imaging to obtain a single image of Optos (O) and the montage of two images of the Clarus (C). The highest number of traceable vessel branches in the four retinal quadrants was determined by two masked raters. An image was classified as “O > C” when the number of identifiable branch was greater in the Optos than the Clarus, as “O = C” when the number was equal and as “O < C” when the number was fewer in the Optos than the Clarus.

**Results:**

The appearance probability of “O > C” was significantly higher at the upper temporal quadrant than “O < C” (*p* < 0.01 for both raters). In contrast, the appearance probability of “O < C” was significantly higher at the lower nasal quadrant than “O > C” (*p* < 0.01 for both raters). There were no significant differences in the appearance probability between “O > C” and “O < C” at the other two retinal quadrants (*p* > 0.50 for both raters).

**Conclusions:**

These results demonstrate that the effective field of views was different between the two devices at different retina quadrants. Further studies are needed to clarify possible factors such as artifacts by the eyelashes, differences in the depth of focus, motion of the device, and different locations of the images on the effective field of views.

## 1. Introduction

Ultra-widefield fundus imaging is being used more often in clinical practices. With this system, a noninvasive retinal image of up to 200° can be obtained without pupillary dilation [1]. In addition, various image modalities can be obtained including fluorescein angiography [2–4], indocyanine green angiography [5], and fundus autofluorescence [6].

A definition of the size of an ultra-widefield fundus image has not been defined. The Diabetic Retinopathy Clinical Research network (DRCRnet) proposed that a fundus photograph with a field of view of ≥100° can be considered to be an ultra-widefield photograph [7]. In contrast, the International Widefield Imaging Study Group recently suggested that the term “ultra-widefield” should be used to describe images showing a single view of the retina of the far periphery of all four quadrants, although they did not propose any specific values of the field of view [8].

One of the most widely used ultra-widefield fundus imaging system is the Optos® 200Tx (Optos, Dunfermline, UK). This device can record a photograph of the fundus up to 200° which is more than 80% of the retina. It uses an ellipsoidal mirror-based confocal scanning laser (cSLO) [1]. The use of this system was quickly incorporated into the clinical practice because it made accurate and rapid widefield images possible without pupillary dilation [9–12]. However, there were some disadvantages of this device including a pseudocolor image using red and green lasers and different magnification between the central and peripheral retinas [13, 14].

In 2017, another type of ultra-widefield retinal imaging system, the Clarus™ 500 (Carl Zeiss Meditech Inc., Dublin, USA), was introduced. This system can obtain a true color fundus image using LEDs emitting red, green, and blue light. Although the viewing angle of the Clarus is 135° when a single image is used, this system is designed to automatically synthesize an ultra-widefield image from two images recorded from different horizontal viewing angles. The manufacturer claimed that the horizontal angle of view of this montage image was 200° which is similar to the size of the Optos image.

Although there is one recent report comparing the images obtained by Optos and Clarus in determining the severity of diabetic retinopathy [15], these two devices have not been directly compared in terms of the degree of visibility of the peripheral retina.

Thus, the purpose of this study was to compare the effective viewing field of view of the Optos and Clarus based on the number of retinal vessel branches that can be detected in the four retinal quadrants.

## 2. Subjects and Methods

### 2.1. Study Design

This was a single-center, retrospective, cross-sectional study of the medical records of patients examined in the Department of Ophthalmology, Mie University Hospital in May, 2018. The procedures used conformed to the tenets of the Declaration of Helsinki of the World Medical Association. All protocols were reviewed and approved by the Ethics Committee of Mie University Hospital (approval number: H2018-043). A written informed consent was not obtained from the subjects because of the retrospective nature of this study. Instead, a home page was created with information on the purpose of this study for the subjects to read. We emphasized that there was a statement in the consent form stating that any subjects could opt out of the study at any time by telephone, fax, or e-mail.

### 2.2. Subjects

The subjects were patients, and normal subjects who visited our hospital and underwent retinal photography by both the Optos and Clarus devices in May of 2018. The pupils were not dilated to record fundus images by both devices. If the subjects were normal and healthy, only one eye was chosen for the analysis, and the eye selected was alternated between the right or left eyes of consecutive patients.

The following cases were excluded: cases in which the identification of vessel branches was difficult due to severe cataract or vitreous opacities; cases in which only a single image of 133° was recorded by the Clarus device; cases whose pupil size was too small to record clear retinal images.

### 2.3. Fundus Imaging by Optos System

**Figure 1:**
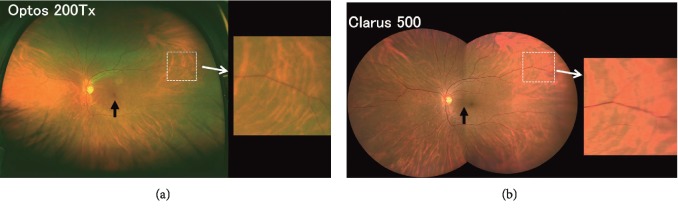
Representative retinal images recorded with a viewing angle of 200° in the horizontal plane by the Optos 200Tx and Clarus 500; two ultra-widefield ophthalmoscopes, from the same patient (normal subject, 40-year-old man). A single image obtained by the Optos (a) and a montage image created by the two images of the Clarus (b) are shown. Magnified views of the area outlined by a dashed white line are shown in the right panels. We noted that the blood vessels of the peripheral retina appear to be more blurred in the Clarus image than the Optos image. Black arrows also indicate the center of the image.

The Optos is a cSLO system that can obtain a retinal image of 200° in one frame [1]. The light sources were a 532 nm green and a 633 nm red laser light. With this system, a single-shot, macula-centered ultra-widefield color fundus image can be obtained without pupillary dilation (Figure 1(a), upper left panel). The time for photographing each image was 0.4 sec. The Optos fundus images are presented in a pseudocolor image of the retina which were balanced for the green and red laser images by an examiner, so that the retinal images were most clearly seen. The fundus images were extracted as JPG files consisting of 3900 × 3072 pixels for further evaluations.

It has been reported that the use of an eyelid-opening device is effective in avoiding eyelash artifacts in the Optos images [16]. However, we did not use this method because we wanted to compare the retinal images of the two devices under the usual clinical conditions.

### 2.4. Fundus Imaging with Clarus System

The light sources of the Clarus were a combination of three colored light emitting diodes (LEDs; red, 585–640 nm; green, 500–585 nm; blue, 435–500 nm). A combination of these three light sources provided a true color fundus image. The fundus image was acquired by a cSLO with partial confocal optics. The photographing time was 0.15 sec. A single image and two fundus images were recorded from two different horizontal visual angles using an internal fixation light. These two images were automatically merged to create a montage image with a 200° field of view (Figure 1, lower left panel). The fundus images were extracted as JPG files consisting of 6604 × 4274 pixels.

### 2.5. Evaluation of Number of Identifiable Vessel Branches

The retinal images obtained by the two devices from the same individual were displayed on a 13″ MacBook Pro with Retina display (Apple Inc., Cupertino, CA) with a standard resolution of 2560 × 1600 pixels (89 pixels/mm) and 500 Nits (=cd/m^2^) brightness. The two raters were permitted to enlarge the images and adjust the color tone and contrast using the Mac Preview application.

**Figure 2:**
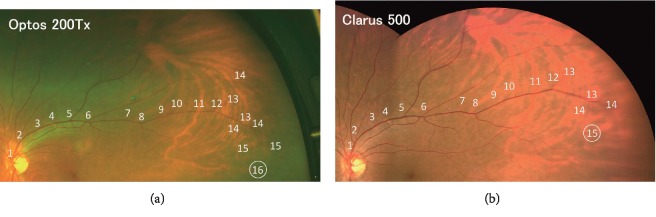
An example of comparing the number of the highest identifiable vessel branches in the upper temporal quadrant, which was performed by Rater 1. The vessel branches are sequentially numbered starting from the optic disc. In this quadrant, the 16th branch of the vessel was the highest number of branches identified in the Optos image (a) whereas the 15th branch of the vessel was the highest number of branch in the Clarus image (b). At this upper temporal quadrant, this image was classified as “O > C” by Rater 1 because the highest number of vessel branches was larger in the Optos image than in the Clarus image.

To evaluate the effective view angles of the two ultra-widefield ophthalmoscopes, the highest number of retinal vessel branches was determined in the four retinal quadrants. For this, the rater was asked to look at one retinal quadrant of the Optos and Clarus images carefully and identify a single retinal vessel which seemed to have the highest number of retinal branches. Then, the rater traced this retinal vessel to the periphery and counted the number of retinal branches on the images from the two ultra-widefield ophthalmoscopes (Figure 2). After the rater confirmed the highest number of retinal vessel branches in the two images in one quadrant, the relative superiority of view angle between the two devices was evaluated. An image was classified as “O > C” when the number of traceable vessel branches was higher in the Optos image than in the Clarus image. It was classified as “O < C” when the traceable vessel branch was higher in the Clarus image than in the Optos image. It was classified as “C = O” when the highest number of branches was equal for the two images. This evaluation was performed at the four retinal quadrants separately and was done by two retina specialists independently (YM and AI).

### 2.6. Statistical Analyses

The level of agreements between the two raters in the classification of the images into “O > C,” “O < C,” or “O = C” was assessed using Kendall's coefficient of concordance [17]. The value of the coefficient of concordance ranges from 0 to 1. It is generally accepted that the inter-rater reliability is fairly high when the coefficient of concordance is >0.75 [18].

Because the manufacturer's claim that the horizontal view angle of image was 200° for both devices, their performances were assumed to be equivalent for the two images. After excluding the cases evaluated as “C = O”, the probability of the appearance of “C > O” and “C < O” was compared by a binomial test. This test was conducted for the two raters for the four quadrants (upper temporal, lower temporal, upper nasal, and lower nasal). The results were considered statistically significant when *p* < 0.05.

## 3. Results

Of the 128 patients who underwent retinal photography by both devices, 90 eyes from 90 patients met the eligibility criteria. The demographics of these patients are presented in Table 1. The mean age of the patients was 60.1 ± 15.8 years with a range of 18 to 85 years. Twenty-one eyes had undergone cataract surgery with intraocular lens implantation, and 9 eyes had a history of vitreous surgery. The subjects included five normal eyes, 31 eyes with cataracts, 21 eyes with glaucoma, 11 eyes with diabetic retinopathy, seven eyes with retinal detachment, and 15 eyes with other retinal diseases. All retinal images of the eyes with retinal detachment were obtained postoperatively.

Representative retinal images recorded from the left eye of a normal subject (40-year-old man) using a viewing angle of 200° in the horizontal plane by the two devices are shown in Figure 1. The color image of the retina was more natural in the Clarus image than the pseudocolor image of the Optos. It can be seen that the inferior parts of the retinal images were blocked by the subject's eyelashes in the Optos image whereas there were only minor blockages in the Clarus image. In addition, we noticed that the blood vessels of the central retina were seen more clearly in the Clarus image than the Optos image. However, the blood vessels of the peripheral retina appeared to be more blurred in the Clarus image than in the Optos image (right panels).

An example of the counting of the number of vessel branches in one quadrant by one rater (Rater 1) is shown in Figure 2. These are enlarged images of the upper temporal quadrant of the same subject shown in Figure 1. The branch positions are sequentially numbered starting from the optic disc. In this quadrant, 16 branches of the vessel were the highest number of branches detected in the Optos image (upper panel). On the other hand, there were 15 branches of the vessel detected in the Clarus image. Based on these results, the upper temporal area of this image was classified as “O > C” by Rater 1.

**Figure 3:**
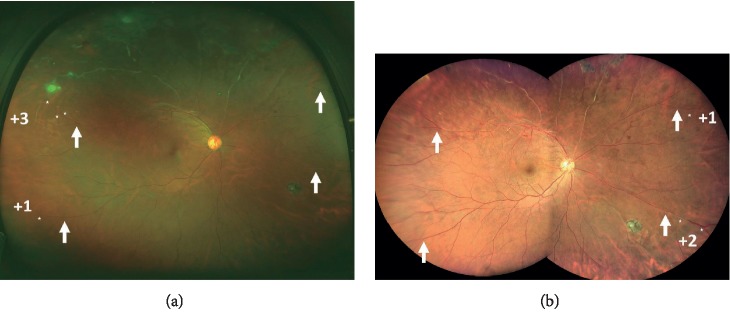
Fundus images obtained by the Optos and Clarus devices of a 42-year-old man with Coats' disease that was evaluated by Rater 2. White arrows indicate the positions of the most peripheral branch identified on both devices. The asterisks indicate the branching positions more peripheral to the arrow in one device. In addition, the numbers indicate the branches identified beyond the most peripheral branch by the other device. In this case, three additional branches were identified in the upper temporal quadrant and an additional branch in the lower temporal quadrant pn the Optos image than the Clarus image. In contrast, when compared with the Optos image, an additional branch was identified in the upper nasal quadrant and two additional branches in the lower nasal quadrant in the Clarus image. Therefore, the upper temporal and lower temporal quadrants were classified as “O > C” and the superior nasal and inferior nasal quadrants as “O < C” in this case by Rater 2.

The results from a representative case of a 42-year-old man with Coats' disease evaluated by one rater (Rater 2) are shown in Figure 3. In this figure, the white arrows point to the positions of the most peripheral branch identified in the images from both devices. The asterisks indicate the branching positions more peripheral to the arrow positions in one device. In addition, the numbers indicate the branches identified beyond the most peripheral branch by the other device. In this case, three additional branches were identifiable at the upper temporal quadrant and an additional branch in the lower temporal quadrant in the Optos image than in the Clarus image. In contrast, an additional branch was identified at the upper nasal quadrant and two additional branches at the lower nasal quadrant in the Clarus image than in the Optos image. Therefore, the upper temporal and lower temporal quadrants were classified as “O > C” and the upper nasal and lower nasal quadrants as “O < C” by Rater 2.

The number of the C > O, C = O, or C < O evaluations of the four retinal quadrants for the two raters is shown in Table 2. At the upper temporal quadrant, the number of identifiable branches in the Optos image was higher than that of Clarus (O > C) in the 41 images for Rater 1 and 35 images for Rater 2. The number of traceable branches on the Optos was equal to that of Clarus (C = O) in 36 images for Rater 1 and 42 images for Rater 2. The number of traceable branches on the Clarus was higher than that of Optos (O < C) in 13 images for two raters. Examination of Table 2 shows that the number of “O > C” tended to be higher than that of “O < C” at the upper temporal quadrant, and the number of “O < C” tended to be higher than that of “O > C” at the lower nasal quadrant.

In the very right column of Table 2, the results of the inter-rater reliability analyses using Kendall's coefficient of concordance are shown. The coefficient of concordance for the two raters ranged from 0.79 to 0.81 at the four quadrants which indicated fairly good agreements between the two raters.

Finally, we performed statistical comparisons of the appearance probabilities between “O > C” and “O < C” after excluding “C = O” (Table 3). The results of the binomial tests showed that the appearance probability of “O > C” was significantly higher at the upper temporal quadrant than that of “O < C” for the two raters (Rater 1, *p* < 0.001; Rater 2, *p*=0.002). In contrast, the appearance probability of “O < C” was significantly higher at the lower nasal quadrant than that of “O > C” for the two raters (Rater 1, *p*=0.002; Rater 2, *p*=0.009). There was no significant difference in the appearance probabilities between “O > C” and “O < C” at the other two retinal quadrants (*p* > 0.5 for two raters).

## 4. Discussion

The results demonstrated that the number of identifiable branches in the Optos images were higher than that in the Clarus images at the upper temporal quadrant, and those in the Clarus were higher than those in the Optos at the lower nasal quadrant (Table 3). In the other two quadrants, there was no significant difference between two devices. The agreement of these findings for the two raters was determined to be fairly good with an inter-rater reliability index of 0.79 to 0.81 (Table 2).

It is difficult to explain why there were such significant differences in the effective view angles between the two devices at the upper temporal and lower nasal quadrants. However, we believe there are at least four possible factors. The first factor is the artifacts caused by the eyelashes. It is known that the inferior parts of retinal image are often blocked by the patient's lashes in the Optos image [1, 4, 16]. In contrast, the Clarus uses partially confocal optics which effectively reduces the artifacts by the eyelids and eyelashes [15] (Figure 1). This may be one of the factors for the better effective view angles for the Clarus in the lower retinal quadrants. Although the partial confocal optics used in the Claus has an advantage by omitting the artifacts from the anterior segments such as eyelashes, it also has a disadvantage of having obscure images of the peripheral retina as will be described.

The second factor is the differences in the depth of focus of the two devices. We noted that the peripheral retinal blood vessels tended to be seen more clearly in the Optos than in the Clarus images (right panels, Figure 1). The depth of focus is wide enough in both devices to view extensive retinal areas from the macula to the peripheral retina. However, the depth of focus of Optos, which is equipped with the cSLO system with an ellipsoidal mirror [1], is wider than that of Clarus. This difference in the degree of depth of focus was supposed to be one reason why peripheral retinal blood vessels tended to be seen more clearly in the Optos images than in the Clarus images especially in the temporal retina.

The third factor is the movement of the camera. In the Optos, the subject's face is pressed against the instrument during the recording of an image, and the device is not moved. In contrast, there is a working distance of 25 mm between the camera and patient's face with the Clarus, and the examiner has to swing the body of the camera right and left when they record two photographs to be able to create a 200° montage image. In the Clarus, therefore, it is necessary to avoid the subject's nose when the examiner photographs the temporal retina while swinging the camera horizontally, whereas there is no such interference when the nasal retina is photographed. As a result, the imaging of the most peripheral area on the temporal side can be slightly more difficult with the Clarus. This may be one of the reasons why the effective view angle of Clarus tended to be narrower than Optos for the temporal retina.

The fourth factor is the different locations of the center of image. In Optos, the center of image corresponds to the fovea while the center of image is located slightly to the nasal retina from the fovea in the montage image of Clarus (black arrows. Figure 1). This difference may be a reason why the effective view angle of Clarus became wider on the nasal side than the temporal side.

Other than these four major differences between the two devices, other factors may be involved including the different light sources, different resolution (Optos, 14 *μ*m; Clarus, 7 *μ*m), different methods of confocal laser scanning, and different methods of creating planar images of the curved fundus surface.

There are two major limitations in this study. First, we compared only the number of identifiable vessel branches in the two devices. Other comparisons of the detection rate of peripheral retinal lesions, grading or progression of retinal diseases [15], and diagnostic power of retinal diseases may be needed to compare the actual clinical usefulness of the ultra-widefield devices in more detail. Second, we compared two retinal images with different number of pixels, viz., 3900 × 3072 pixels vs. 6604 × 4274 pixels. This difference might have affected the results.

## 5. Conclusions

We compared the effective view angles of two ultra-widefield ophthalmoscopes, Optos and Clarus, based on the number of identifiable retinal vessel branches in the four retinal quadrants. We found that the effective view angle was wider in the Optos in the upper temporal periphery and was wider in the lower nasal periphery in the Clarus. Several optical and structural factors in the two devices seem to be involved in the difference in effective view angles at the different retinal locations.

## Figures and Tables

**Table 1:** Demographic data of 90 eyes of 90 patients who underwent retinal photography by both Optos® 200Tx and Clarus™ 500.

Parameter	Value
Number of eyes/subjects	90/90
Age, mean ± SD, year	60.1 ± 15.8
Sex	
Men	59
Women	31
Lens	
Phakic	69
Pseudophakic	21
Vitreous	
Vitreous	81
Avitreous	9
Disease	
Cataract	31
Glaucoma	21
Diabetic retinopathy	11
Rhegmatogenous retinal detachment	7
Uveitis	3
Retinal vein occlusion	3
Age-related macular degeneration	3
Macular dystrophy	2
Macular hole	1
Central serous chorioretinopathy	1
Epiretinal membrane	1
Coat's disease	1
Normal subject	5

**Table 2:** Number of three gradings, C > O, C = O, or C < O based on the highest number of traceable vessel branch by the two raters in the four retinal quadrants.

	Number of “O > C”	Number of “O = C”	Number of “O < C”	Kendall's coefficient of concordance
Upper temporal (Rater 1/Rater 2)	41/35	36/42	13/13	0.79
Lower temporal (Rater 1/Rater 2)	27/22	41/49	22/19	0.79
Upper nasal (Rater 1/Rater 2)	22/20	48/55	20/15	0.81
Lower nasal (Rater 1/Rater 2)	12/11	45/51	33/28	0.79

An image was graded as “O > C” when the highest number of traceable vessel branch was larger in the Optos image than in the Clarus image. An image was graded as “O < C” when the highest number of traceable vessel branch was larger on the Clarus image than on the Optos image. An image was graded as “O = C” when the highest number of traceable vessel branch was equal in the Optos and Clarus images. Inter-rater reliability in the grading of the image into “O > C,” “O < C,” or “O = C” by the two raters was assessed using Kendall's coefficient of concordance.

**Table 3:** Comparison of appearance probability between “O > C” and “O < C” at four retinal quadrants by two raters.

		Appearance probability of “O > C” (95% C.I.)	Appearance probability of “O < C” (95% C.I.)	*p* value
Upper temporal	Rater 1	75.9 (62.4–86.5)	24.1 (13.5–37.6)	<0.001^*∗*^
	Rater 2	72.9 (58.2–84.7)	27.1 (15.3–41.8)	0.002^*∗*^

Lower temporal	Rater 1	55.1 (40.2–69.3)	44.9 (30.7–59.8)	0.568
	Rater 2	53.7 (37.4–69.3)	46.3 (30.7–62.6)	0.755

Upper nasal	Rater 1	52.4 (36.4–68.0)	47.6 (32.0–63.6)	0.878
	Rater 2	57.1 (39.4–73.7)	42.9 (26.3–60.6)	0.500

Lower nasal	Rater 1	26.7 (14.6–41.9)	73.3 (58.1–85.4)	0.002^*∗*^
	Rater 2	28.2 (15.0–44.9)	71.8 (55.1–85.0)	0.009^*∗*^

Statistical comparisons were performed by binomial tests. The results were considered statistically significant when *p* < 0.05. C.I., confidential interval.

## Data Availability

The raw data used to support the findings of this study are added in the Supplementary Materials.

## Supplementary Materials

Supplementary Materials Results of relative superiority of view angle between the two devices for 90 subjects. After two raters (Rater 1 and Rater 2) identified the highest number of retinal vessel branches in one quadrant, the relative superiority of view angle between the two devices was evaluated. An image was classified as “O > C” (=2) when the number of traceable vessel branches was higher in the Optos image than the Clarus image. It was classified as “O < C” (=0) when the traceable vessel branch was higher in the Clarus image than in the Optos image. It was classified as “C = O” (=1) when the highest number of branches was equal for the two images.
