# Supplementary material for: Comparisons of Effective Fields of Two Ultra-Widefield Ophthalmoscopes, Optos 200Tx and Clarus 500
Source: Biomed Res Int. 2019 Dec 5;2019:7436293. doi: 10.1155/2019/7436293 (PMC6915147; doi:10.1155/2019/7436293)
Supplement: Supplementary Materials — Results of relative superiority of view angle between the two devices for 90 subjects. After two raters (Rater 1 and Rater 2) identified the highest number of retinal vessel branches in one quadrant, the relative superiority of view angle between the two devices was evaluated. An image was classified as “O > C” (=2) when the number of traceable vessel branches was higher in the Optos image than the Clarus image. It was classified as “O < C” (=0) when the traceable vessel branch was higher in the Clarus image than in the Optos image. It was classified as “C = O” (=1) when the highest number of branches was equal for the two images. [file 7436293.f1.docx]

| **Subject ID** | **Upper-temporal** | | **Lower-temporal** | | **Upper-nasal** | | **Lower-nasal** | |
| --- | --- | --- | --- | --- | --- | --- | --- | --- |
|  | **Rater 1** | **Rater 2** | **Rater 1** | **Rater 2** | **Rater 1** | **Rater 2** | **Rater 1** | **Rater 2** |
| **1** | 2 | 1 | 1 | 1 | 1 | 1 | 1 | 1 |
| **2** | 2 | 1 | 1 | 1 | 1 | 1 | 1 | 2 |
| **3** | 2 | 2 | 2 | 1 | 2 | 2 | 1 | 1 |
| **4** | 0 | 0 | 1 | 1 | 2 | 1 | 1 | 2 |
| **5** | 2 | 1 | 1 | 1 | 2 | 2 | 1 | 0 |
| **6** | 2 | 0 | 1 | 1 | 1 | 0 | 1 | 1 |
| **7** | 0 | 1 | 1 | 0 | 0 | 0 | 0 | 0 |
| **8** | 1 | 0 | 2 | 1 | 1 | 2 | 1 | 1 |
| **9** | 2 | 2 | 2 | 2 | 2 | 2 | 1 | 1 |
| **10** | 1 | 1 | 2 | 1 | 1 | 1 | 1 | 1 |
| **11** | 0 | 1 | 1 | 1 | 0 | 0 | 0 | 0 |
| **12** | 1 | 1 | 2 | 1 | 0 | 0 | 0 | 1 |
| **13** | 1 | 1 | 1 | 1 | 2 | 2 | 0 | 1 |
| **14** | 1 | 1 | 1 | 1 | 1 | 1 | 0 | 1 |
| **15** | 2 | 1 | 2 | 2 | 1 | 1 | 1 | 1 |
| **16** | 1 | 2 | 1 | 2 | 2 | 2 | 2 | 2 |
| **17** | 1 | 1 | 1 | 1 | 1 | 1 | 0 | 0 |
| **18** | 2 | 2 | 1 | 1 | 1 | 1 | 1 | 1 |
| **19** | 0 | 1 | 2 | 1 | 0 | 2 | 1 | 1 |
| **20** | 2 | 1 | 2 | 2 | 1 | 1 | 1 | 1 |
| **21** | 1 | 1 | 1 | 1 | 1 | 1 | 0 | 1 |
| **22** | 1 | 1 | 2 | 2 | 1 | 1 | 0 | 0 |
| **23** | 2 | 2 | 1 | 2 | 1 | 1 | 1 | 0 |
| **24** | 2 | 1 | 2 | 1 | 1 | 2 | 0 | 1 |
| **25** | 2 | 2 | 1 | 2 | 1 | 2 | 1 | 1 |
| **26** | 2 | 1 | 2 | 2 | 1 | 1 | 2 | 1 |
| **27** | 2 | 2 | 1 | 1 | 0 | 1 | 0 | 1 |
| **28** | 1 | 1 | 0 | 0 | 1 | 1 | 1 | 0 |
| **29** | 2 | 2 | 2 | 1 | 1 | 1 | 1 | 1 |
| **30** | 1 | 2 | 1 | 1 | 1 | 1 | 1 | 0 |
| **31** | 1 | 1 | 2 | 2 | 1 | 1 | 1 | 1 |
| **32** | 1 | 2 | 2 | 2 | 1 | 1 | 1 | 1 |
| **33** | 1 | 1 | 2 | 1 | 1 | 1 | 1 | 1 |
| **34** | 1 | 1 | 1 | 1 | 1 | 1 | 0 | 0 |
| **35** | 2 | 2 | 2 | 2 | 1 | 1 | 0 | 1 |
| **36** | 2 | 1 | 2 | 1 | 2 | 1 | 0 | 2 |
| **37** | 1 | 2 | 1 | 1 | 1 | 1 | 1 | 1 |
| **38** | 2 | 2 | 2 | 2 | 1 | 1 | 1 | 1 |
| **39** | 2 | 2 | 2 | 2 | 0 | 1 | 0 | 0 |
| **40** | 2 | 2 | 0 | 1 | 2 | 2 | 2 | 1 |
| **41** | 0 | 0 | 0 | 0 | 0 | 1 | 1 | 1 |
| **42** | 1 | 1 | 1 | 1 | 1 | 1 | 1 | 1 |
| **43** | 1 | 1 | 1 | 0 | 1 | 1 | 1 | 1 |
| **44** | 2 | 1 | 2 | 2 | 0 | 1 | 2 | 1 |
| **45** | 2 | 2 | 1 | 2 | 1 | 0 | 1 | 1 |
| **46** | 1 | 0 | 1 | 1 | 1 | 1 | 0 | 0 |
| **47** | 2 | 1 | 1 | 1 | 1 | 1 | 1 | 1 |
| **48** | 1 | 1 | 0 | 0 | 1 | 0 | 1 | 1 |
| **49** | 2 | 2 | 0 | 0 | 1 | 1 | 1 | 1 |
| **50** | 2 | 2 | 0 | 1 | 1 | 1 | 2 | 2 |
| **51** | 1 | 1 | 0 | 0 | 1 | 1 | 0 | 0 |
| **52** | 2 | 2 | 2 | 1 | 0 | 1 | 0 | 1 |
| **53** | 1 | 1 | 0 | 1 | 1 | 1 | 1 | 1 |
| **54** | 2 | 1 | 1 | 1 | 2 | 2 | 1 | 1 |
| **55** | 2 | 2 | 1 | 1 | 2 | 2 | 1 | 1 |
| **56** | 0 | 1 | 1 | 1 | 2 | 1 | 1 | 1 |
| **57** | 2 | 2 | 0 | 1 | 0 | 1 | 2 | 2 |
| **58** | 2 | 2 | 2 | 1 | 2 | 1 | 0 | 1 |
| **59** | 1 | 2 | 2 | 2 | 2 | 2 | 1 | 1 |
| **60** | 0 | 0 | 0 | 0 | 0 | 0 | 0 | 0 |
| **61** | 2 | 2 | 2 | 2 | 2 | 2 | 2 | 2 |
| **62** | 2 | 1 | 1 | 1 | 2 | 1 | 1 | 1 |
| **63** | 1 | 0 | 0 | 1 | 1 | 1 | 0 | 0 |
| **64** | 1 | 1 | 1 | 0 | 2 | 1 | 0 | 0 |
| **65** | 1 | 2 | 1 | 1 | 1 | 1 | 0 | 0 |
| **66** | 2 | 1 | 1 | 0 | 1 | 1 | 1 | 1 |
| **67** | 2 | 2 | 0 | 2 | 0 | 0 | 2 | 1 |
| **68** | 0 | 0 | 1 | 1 | 2 | 1 | 0 | 0 |
| **69** | 1 | 1 | 1 | 0 | 1 | 1 | 1 | 0 |
| **70** | 2 | 2 | 1 | 1 | 2 | 2 | 2 | 2 |
| **71** | 2 | 2 | 1 | 2 | 2 | 2 | 0 | 1 |
| **72** | 1 | 2 | 0 | 0 | 1 | 1 | 2 | 0 |
| **73** | 2 | 2 | 2 | 2 | 1 | 0 | 1 | 1 |
| **74** | 0 | 0 | 0 | 1 | 1 | 2 | 0 | 0 |
| **75** | 2 | 1 | 2 | 2 | 1 | 2 | 2 | 2 |
| **76** | 2 | 2 | 1 | 1 | 1 | 1 | 1 | 2 |
| **77** | 1 | 2 | 0 | 1 | 1 | 1 | 0 | 0 |
| **78** | 1 | 1 | 0 | 1 | 0 | 0 | 0 | 0 |
| **79** | 2 | 2 | 0 | 1 | 1 | 1 | 2 | 2 |
| **80** | 2 | 2 | 0 | 0 | 1 | 1 | 1 | 1 |
| **81** | 1 | 1 | 1 | 1 | 0 | 0 | 0 | 0 |
| **82** | 1 | 2 | 1 | 1 | 2 | 2 | 1 | 1 |
| **83** | 1 | 1 | 1 | 0 | 0 | 0 | 0 | 0 |
| **84** | 0 | 0 | 1 | 0 | 0 | 1 | 1 | 0 |
| **85** | 1 | 1 | 0 | 0 | 2 | 1 | 1 | 1 |
| **86** | 0 | 0 | 2 | 2 | 0 | 0 | 0 | 1 |
| **87** | 1 | 1 | 0 | 0 | 0 | 0 | 0 | 0 |
| **88** | 0 | 0 | 0 | 0 | 2 | 2 | 1 | 1 |
| **89** | 1 | 0 | 1 | 0 | 0 | 1 | 0 | 0 |
| **90** | 0 | 1 | 0 | 1 | 0 | 0 | 0 | 0 |

**Supplementary material**

Results of relative superiority of view angle between the two devices for 90 subjects. After two raters (rater 1 and rater 2) identified the highest number of retinal vessel branches in one quadrant, the relative superiority of view angle between the two devices was evaluated. An image was classified as ‘‘O>C’’ (=2) when the number of traceable vessel branches was higher in the Optos image than the Clarus image. It was classified as ‘‘O<C’’ (=0) when the traceable vessel branch was higher in the Clarus image than in the Optos image. It was classified as ‘‘C=O’’ (=1) when the highest number of branches was equal for the two images.
